# Boosting entrepreneurial intentions: A novel EEG-guided protocol

**DOI:** 10.1016/j.mex.2023.102358

**Published:** 2023-09-01

**Authors:** Bhairab Chandra Patra, Dibya Nandan Mishra

**Affiliations:** a Jindal Global Business School, O. P. Jindal Global University, Sonipat, India; b Symbiosis Institute of Business Management Pune, Symbiosis International (Deemed University), Pune, India

**Keywords:** Entrepreneur, EEG, Human behavior, Cognitive mapping, Entrepreneurial Intention Development System

## Abstract

This research proposal aims to explore the driving factors of an entrepreneurial mindset and develop pedagogies to boost entrepreneurial intention (EI) in entrepreneurially unmotivated individuals. Using an electroencephalogram (EEG) cap, the study will map the cognitive processes associated with entrepreneurship in both entrepreneurially motivated and unmotivated individuals. The expected outcomes include identifying crucial factors for a positive entrepreneurial intention and discovering effective teaching methodologies. The implications of this research extend to educational institutions, policy-making bodies, and incubation centers, supporting initiatives such as Start-up India and Stand-up India. Overall, this study contributes to entrepreneurship education and practical training for individuals lacking motivation.

## Graphical abstract

**Figure:**
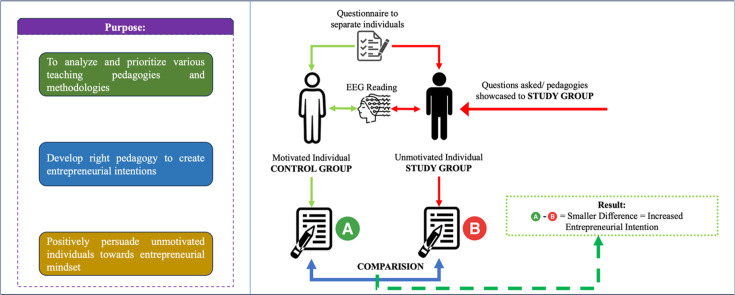

Specifications table

**Table:** 

Subject Area:	Psychology
More specific subject area:	Cognitive Psychology
Protocol name:	*Entrepreneurial Intention Development System*
Reagents/tools:	*Open BCI Ultracortex Biosensing Headset. Manufacturer – OpenBCI. Product specification – 8/16 Channel KIT.*
Experimental design:	This research involves recording EEG signals of these two different populations using 8/16 channel EEG. The control group consists of individuals with significant entrepreneurial motivation/intention, while the study group comprises individuals lacking such motivation. Participants will be selected based on a questionnaire. Before the EEG recording, participants will be instructed to abstain from psychoactive food or drinks for three hours. They will be asked to relax in the recording station for 10–15 min before the recording sessions, during which 5–10 min of EEG signals will be recorded. Then the research will proceed and apply various pedagogies to develop entrepreneurial intention of unmotivated individuals. EEG cap will be used for understanding changes of motivation level after application of each driving methodology.
Trial registration:	*NA*
Ethics:	*Approval from Research Ethical Committee of Woxsen University, Hyderabad, India.*
Value of the Protocol:	• This research protocol seeks to identify the driving factors behind entrepreneurial intentions and develop effective methodologies to motivate individuals towards an entrepreneurial mindset.
	• The studies conducted using this protocol may aim to develop empirically validated methodologies that can be customized to cater to diverse groups of individuals, taking into consideration their demographics and personalities. • The outcomes of this research have implications for policy makers, as the developed protocol can be utilized for training students and individuals to become entrepreneurs, thereby supporting startup initiatives and fostering entrepreneurial ecosystems.

## Description of protocol

Researchers and educationalists often ask, “How can we develop the intention of an individual towards becoming an entrepreneur? What teaching methodologies can be applied and are the most effective?”. Entrepreneurial skills bring in the critical thinking abilities, independent thinking, accepting and overcoming challenges and creativity [3], [4], [5], [6]. Even if individuals do not start their own entrepreneurial journey, the skills of entrepreneurship can help them develop new ideas to solve business problems in a smarter way in their organizations.

A study of management graduates across universities found that not all students develop the critical thinking and problem-solving abilities. In contrast it is found that students with higher intention to become entrepreneurs developed better problem-solving abilities as compared to their other counterparts [1,2,7].

This protocol will help analyze and prioritize various teaching pedagogies and methodologies which may help in developing right pedagogy to create entrepreneurial intentions among unmotivated individual with varying personal skills inclinations and personality types.

The protocol involves recording 8/16 channel EEG signals from one of the two different populations. The first population, considered the control group, consists of participants with significant entrepreneurial motivation/intention. The second population, known as the study group, consists of participants without significant entrepreneurial motivation/intention. The initial classification of participants into these groups is based on a questionnaire survey.

Before the EEG recording, the participants will be informed about the time and place of the recording session. They will be asked to show up at the recording studio at least 30 min before the time allotted. The participants will be asked to refrain from ingesting any food or beverages having psychoactive effects for at least three hours before the recording in order to guarantee reliable findings.

The participants will be invited to relax at the recording station for at least 10 to 15 min when they arrive. Prior to the recording sessions, this relaxation phase seeks to create a tranquil condition. The EEG signals will be recorded during each session, which lasts 5 to 10 min.

The participants will be required to provide their agreement before the EEG recording starts, attesting to their lack of a history of mental illness and agreeing that the administrators are not liable for any medical problems that could develop over the project. A history of mental illness or conditions connected to it will disqualify participants. Since the EEG test is non-invasive, participants won't experience any pain or discomfort, and no professional medical supervision is required.

The administrators will take each participant's head measurements to establish where the EEG electrodes should be placed. The day before the recording session, participants will be instructed to wash their hair, and they will also be told to abstain from alcohol and caffeine for at least eight hours before the test.

**Fig. 1:**
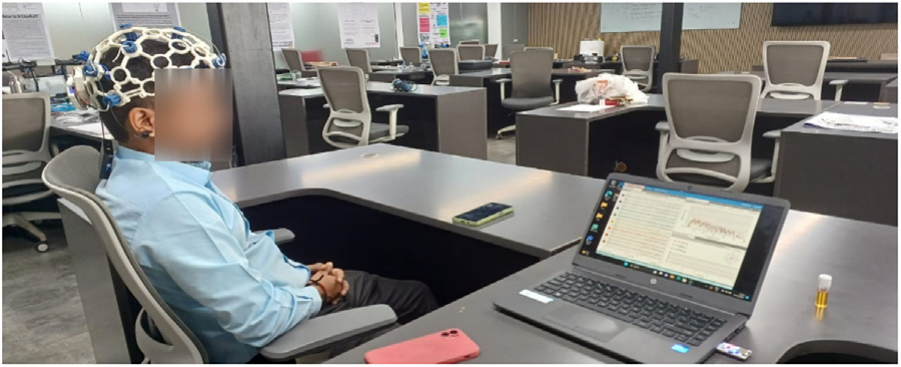
Individual wearing EEG (Side View).

**Fig. 2:**
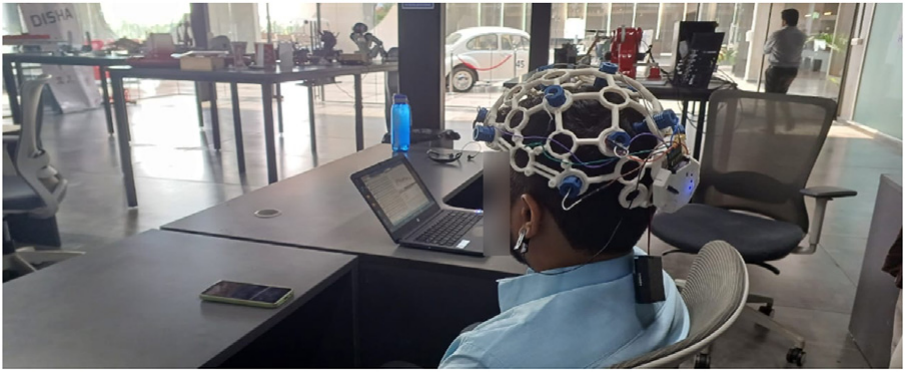
Individual wearing EEG (Back View).

**Fig. 3:**
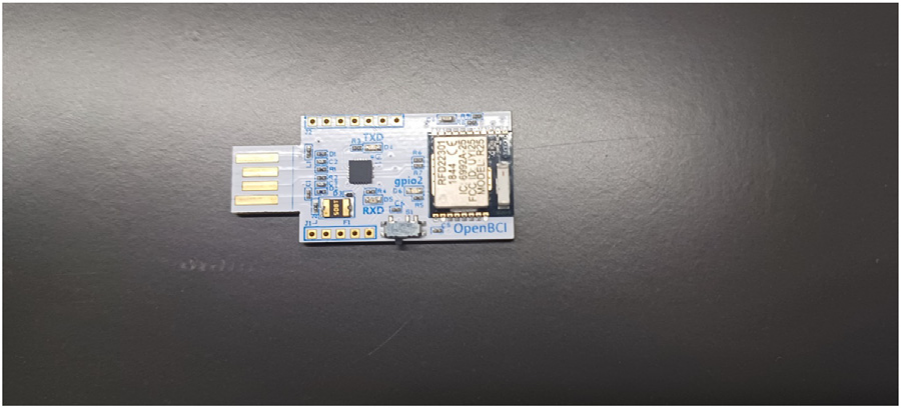
Wireless Connection dongle.

**Fig. 4:**
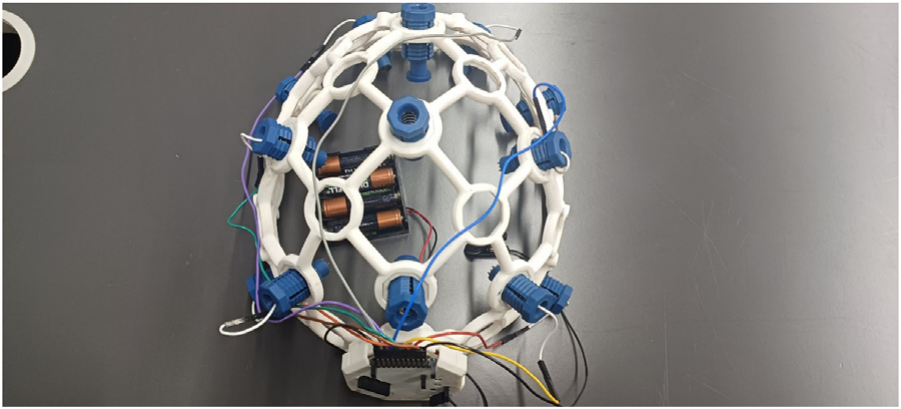
Wireless EEG Cap.

**Fig. 5:**
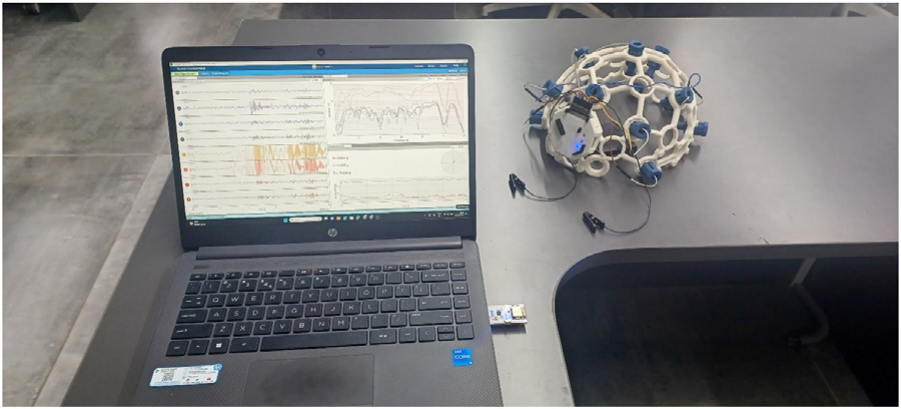
Complete EEG Set.

To ensure the safety and effectiveness of the EEG instruments, the administrators conducted a preliminary session in a controlled environment. No pain or discomfort was observed during or after the experiment. Fig. 1, Fig. 2, Fig. 3, Fig. 4, Fig. 5, provide a visual representation of the experimental setup. Overall, these steps outline the process of recording EEG signals from the control and study groups, ensuring proper participant preparation, comfort, and safety throughout the study.

The EEG study output will be compared between the controlled and the study group, if the difference between the two groups are significantly reduced we can conclude the particular/ group of pedagogies helped increase the entrepreneurial intentions in unmotivated individuals.

## Ethics statement

The authors got approval from the research ethical committee at Woxsen University, Hyderabad, India with ethics approval number WoU/R&D/REC/01053.

## Declaration of Competing Interest

The authors declare that they have no known competing financial interests or personal relationships that could have appeared to influence the work reported in this paper.

## Data Availability

The authors do not have permission to share data.
